# Effect of temperature-humidity index on the evolution of trade-offs between fertility and production in dairy cattle

**DOI:** 10.1186/s12711-024-00889-4

**Published:** 2024-03-29

**Authors:** Aurélie Vinet, Sophie Mattalia, Roxane Vallée, Christine Bertrand, Anne Barbat, Julie Promp, Beatriz C. D. Cuyabano, Didier Boichard

**Affiliations:** 1 Université Paris Saclay, INRAE, AgroParisTech, GABI, 78350 Jouy-en-Josas, France; 2 Institut de L’Elevage, 75012 Paris, France; 3 INRAE, US310 CTIG, Domaine de Vilvert, 78350 Jouy-en-Josas, France

## Abstract

**Background:**

In the current context of climate change, livestock production faces many challenges to improve the sustainability of systems. Dairy farming, in particular, must find ways to select animals that will be able to achieve sufficient overall production while maintaining their reproductive ability in environments with increasing temperatures. With future forecasted climate conditions in mind, this study used data from Holstein and Montbeliarde dairy cattle to: (1) estimate the genetic-by-temperature-humidity index (THI) interactions for female fertility, and (2) evaluate the production-fertility trade-off with increasing values of THI.

**Results:**

Two-trait random regression models were fitted for conception rate (fertility) and test-day protein yield (production). For fertility, genetic correlations between different THI values were generally above 0.75, suggesting weak genotype-by-THI interactions for conception rate in both breeds. However, the genetic correlations between the conception rate breeding values at the current average THI (THI = 50, corresponding to a 24-h average temperature of 8 °C at 50% relative humidity) and their slopes (i.e., potential reranking) for heat stress scenarios (THI > 70), were different for each breed. For Montbeliarde, this correlation tended to be positive (i.e., overall the best reproducers are less affected by heat stress), whereas for Holstein it was approximately zero. Finally, our results indicated a weak antagonism between production and fertility, although for Montbeliarde this antagonism intensified with increasing THI.

**Conclusions:**

Within the range of weather conditions studied, increasing temperatures are not expected to exacerbate the fertility-production trade-off. However, our results indicated that the animals with the best breeding values for production today will be the most affected by temperature increases, both in terms of fertility and production. Nonetheless, these animals should remain among the most productive ones during heat waves. For Montbeliarde, the current selection program for fertility seems to be adequate for ensuring the adaptation of fertility traits to temperature increases, without adverse effects on production. Such a conclusion cannot be drawn for Holstein. In the future, the incorporation of a heat tolerance index into dairy cattle breeding programs would be valuable to promote the selection of animals adapted to future climate conditions.

## Background

The negative effect of heat stress (measured by the temperature-humidity index, or THI) on various traits in dairy cattle has been consistently reported by numerous studies, especially with respect to traits related to production [1–3], reproduction [1, 2, 4], and udder health [1, 5]. Several studies have estimated genotype-by-THI interactions, usually with reaction norm models, that describe the trajectory of genetic parameters along a continuous THI gradient [6, 7]. Nearly systematically, these studies have predicted an unfavorable genetic correlation between the trait level in standard climate conditions and the slope in heat stress conditions. For instance, Vinet et al. [8] found that this correlation was negative for production (i.e., a stronger decline for high-yielding cows) and positive for somatic cell score (i.e., sensitive cows are even more sensitive at high THI). For reproduction, fertility has also been predicted to decline with increasing THI, e.g., [1, 9, 10], which is consistent with in vitro tests (reviewed in [11, 12]). Heat stress is known to adversely affect different stages of cattle reproduction, suppressing the dominance of the large follicle, impairing oocyte quality and embryo development, and increasing embryo mortality (reviewed in [12]). However, data on genotype-by-THI interactions for fertility are still scarce. The estimation of genetic correlations between fertility at different THI values is important, because such correlations provide information about the relevance of current selection goals and their suitability for future forecasted climate conditions, in which higher average THI values are expected.

Irrespective of climate conditions, dairy cows have a physiological need to balance their investment in production with that in other functional traits, a trade-off that is known to generate moderate negative genetic correlations between production and fertility traits [13–15]. This unfavorable genetic correlation has long been incorporated by most countries in their breeding objectives. However, most research on the production-fertility trade-off has occurred in the context of current climate conditions (intermediate average THI), and it is unknown to what extent genetic correlations between production and reproduction traits might depend on environmental factors. Given global forecasts of increasing temperatures, this question has taken on considerable urgency. If correlations between production and reproduction become even more unfavorable at higher THI values, the current selection objectives would again lead to a decrease in fertility. Consequently, the weight of the fertility in the total merit index would have to be increased to prevent such a decline. With future forecasted climate conditions in mind, the aim of this study was to use data from Holstein and Montbeliarde dairy cattle to: (1) estimate genetic-by-THI interactions for female fertility, and (2) evaluate the production-fertility trade-off with increasing values of THI.

## Methods

### Dataset

Performance and pedigree records from 2010 to 2020 were extracted from the French national database hosted by INRAE-CTIG. The fertility phenotype considered was the outcome of the first insemination after first calving, and the production phenotype consisted of test-day protein yield (PY) records from the first lactation, for both Holstein (HOL) and Montbeliarde (MON) cows. The fertility phenotype, hereafter called conception rate (CR), was determined as in Barbat et al. [16]: the outcome was set to 100 (successful) if the cow calved following the expected gestation length of the breed (281 and 285 ± 15 days in Holstein and Montbeliarde, respectively), and 0 otherwise. Insemination data of cows culled before 250 days in milk, i.e. with uncertain status, were excluded. As inseminations with male-sexed semen were very limited and not randomly distributed among lactating cows, these were also discarded. We considered only purebred inseminations performed between 25 and 48 months of age and occurring between 50 and 180 days after calving (more than 90% of the first service records in both breeds). For PY, we collected test-day records occurring between 80 and 200 days of lactation from cows who calved between 23 and 42 months of age. This restriction on days in milk was intended to simplify the random regression model and will be described in further detail below. Cows with unknown parents were removed. The contemporary groups were herd-year (HY) and herd-test-day (HTD) for CR and PY, respectively, and data from contemporary groups with less than three cows were discarded. Only sires with at least 15 daughters with both performances, PY and CR, were retained. The final datasets comprised 3,351,068 (HOL) and 649,814 (MON) CR records, and 10,245,692 (HOL) and 1,966,985 (MON) PY records. These performance data were recorded from a total of 3,368,605 (HOL) and 656,164 (MON) cows, born from 5463 and 1612 sires, respectively. The pedigree of sires was traced back three generations. After trimming, the HOL and MON pedigree files comprised 12,741 and 4148 animals, respectively. Statistics on the raw data are in Table 1.

**Table 1:** Number of cows and records, number of sires, means and standard deviations (sd) of conception rates (CR) and protein yields (PY), and means and sd of average temperature-humidity index (THI) by trait in Holstein and Montbeliarde

	Holstein	Montbeliarde
Number of phenotyped cows	3,368,605	656,164
Number of herds	35,579	9850
Number of CR records	3,351,068	649,814
Number of PY records	10,245,692	1,966,985
Number of sires	5463	1612
Average number of daughters per sire (min–max)	617 (26–49,961)	407 (23–24,884)
Average CR % (sd)	44.5 (49.7)	54.6 (49.8)
Average PY g/d (sd)	839 (176)	740 (164)
Average THIf (sd)	51.3 (8.9)	47.4 (10.6)
Average THIp (sd)	50.7 (9.1)	47.3 (10.7)

THIp average THI in the three days leading up to the test day; THIf average THI in the seven days following insemination

**Fig. 1:**
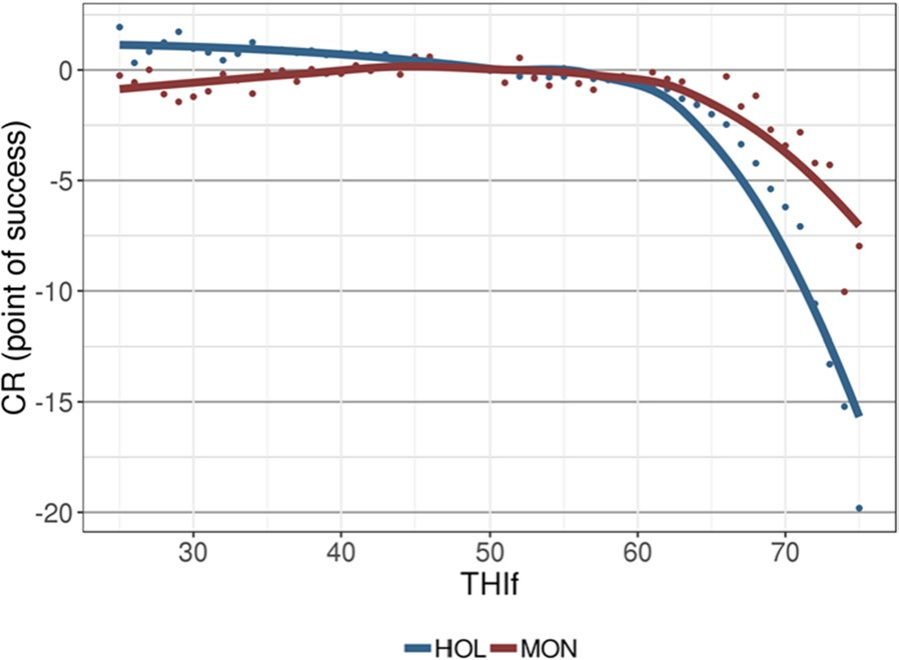
Average effect of average temperature-humidity indices (THIf) on conception rate. Results are given in number of points of success of the insemination compared to the effect at THIf = 50, for the two breeds, Holstein (HOL, in blue) and Montbeliarde (MON, in brown)

**Fig. 2:**
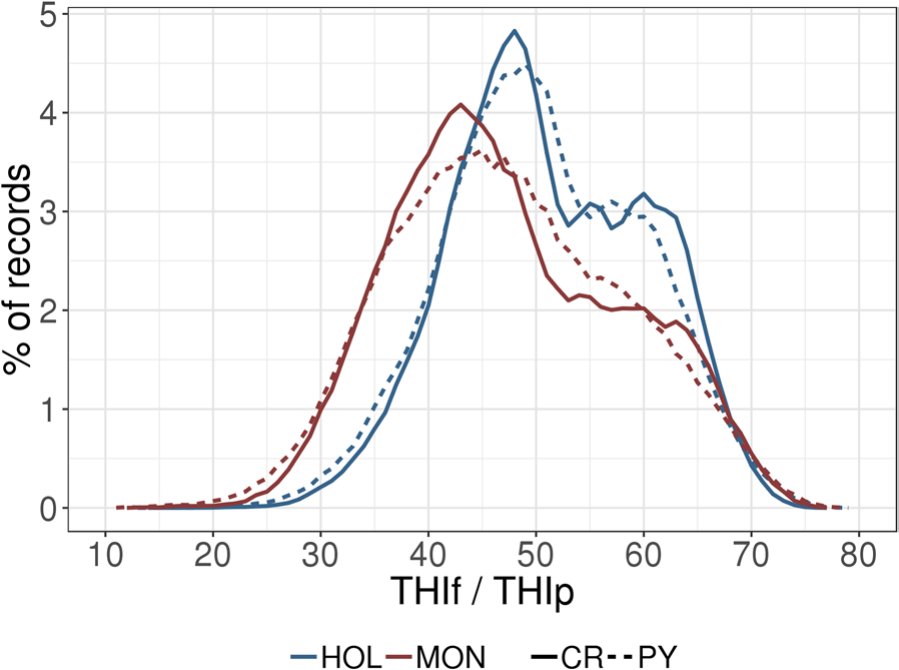
Distribution of average temperature-humidity indices (THI) associated with performance records. Results are given as a percentage of the total records, within-trait and within-breed records. THI values used for the production trait (i.e., protein yield = THIp) are shown with a dashed line and THI values used for the fertility trait (i.e., conception rate = THIf) are shown with a solid line

As in Vinet et al. [8], we used meteorological SAFRAN data provided by the French national meteorological agency (MeteoFrance) that included daily estimates of various indicators for each of the 9892 8 × 8-km^2^ squares that cover the entire French territory. Following the recommendations of Mbuthia et al. [17], we used modeled data instead of observed data. These daily estimates are the result of a model developed for meteorological forecasting based on continuous local recording from a wide network of meteorological stations. The meteorological variables used in this study were the average daily temperature and relative humidity. Based on its zip code, each herd was assigned daily meteorological data consisting of weighted averages of the values from each of the 8 × 8-km^2^ squares overlaying its location, with the weights reflecting the proportion of the area of the village located in each square. The daily THI [18] was calculated as THI = (1.8 × T + 32)−(0.55–0.0055 × RH) × (1.8 × T-26), where T is the 24-h average temperature (Celsius), and RH is the 24-h average relative humidity (%), as adopted in Bohlouli et al. [19] and Brügemann et al. [20]. Two different averages for THI were considered depending on the trait. For PY, THI was averaged over the three days leading up to the test day (i.e., the date of the test and the two previous days) as described in Vinet et al. [8]; this was designated THIp. For CR, we averaged THI over the eight days following insemination (i.e., day of insemination to day 7 after insemination) and denoted this THIf. In preliminary work, we tested several periods between 30 days before and 30 days after insemination (THIf included) and, as in Brügemann et al. [21], we found that different periods provided similar results when analyzed separately because they are highly correlated. The effect of this THIf on conception rate is illustrated in Fig. 1. We chose to use THIf for two reasons: (a) its effect was the most pronounced, and (b) in a model including THI both before and after insemination, the effect of THI before insemination was cancelled out (data not shown), indicating that THI after insemination is more relevant. Indeed, although heat stress affects all reproductive processes of cows (estrus behavior, oocyte quality, pregnancy losses; reviewed in [22]), only the result of insemination (i.e., insemination failure and embryo losses) can be investigated with our data. Figure 2 depicts the distribution of the meteorological variables experienced by herds during the period examined for this study.

### Model for analysis

To estimate genotype-by-THI interactions within traits and the evolution of genetic correlations between traits across various THI conditions, bivariate random regression models were used. Random regression models enable prediction of the performance of each genotype under a given environmental condition. The main objective was to measure the trend in the genetic correlation between CR and PY along a broad THI gradient. Because CR has a very low heritability, very large datasets are required to accurately estimate the varying genetic correlations over the THI gradient. Using a random regression model applied to millions of records, as needed to accurately estimate the trends in genetic correlations over the THI gradient, poses huge computational constraints when applied to an animal model. Therefore, we opted for a sire model, which made it computationally feasible to perform the study that handled all the performance data spanning a wide range of THI conditions. In addition, since each cow had only one CR record, a sire model was deemed more appropriate for describing the effect of the THI gradient in the progeny group.

For test-day production records, it is generally recommended to use a random regression model to account for the varying genetic determinism of PY along the lactation. In previous work by our group [8], we presented a two-dimensional random regression model with components that depended on both days in milk (DIM) and THI. Here, with a CR-PY bivariate approach and a very large dataset, we preferred to avoid this complexity, and instead focused on PY records in the middle of the lactation (80–200 DIM), when genetic parameters are fairly stable [6, 23, 24]. Therefore, no random component associated with DIM was considered.

The following models were used for CR and PY, respectively:

1 $${{\text{y}}}_{{{\text{f}}}_{{\text{i}}}}={\mathbf{x}}_{{\mathbf{f}}_{\mathbf{i}}}{\varvec{\upbeta}}_{\mathbf{f}}+\sum\nolimits_{{\text{k}}=0}^{2}{{\text{z}}}_{{{\text{f}}}_{{\text{jk}}}} {{\text{a}}}_{{{\text{f}}}_{{\text{s}}\left({\text{i}}\right){\text{k}}}} +{{\text{e}}}_{{{\text{f}}}_{{\text{i}}},}$$

2 $${{\text{y}}}_{{{\text{p}}}_{{\text{in}}}}={\mathbf{x}}_{{\mathbf{p}}_{\mathbf{i}\mathbf{n}}}{\varvec{\upbeta}}_{\mathbf{p}}+\sum\nolimits_{{\text{k}}=0}^{3}{{\text{z}}}_{{{\text{p}}}_{{\text{jkn}}}} {{\text{a}}}_{{{\text{p}}}_{{\text{s}}\left({\text{i}}\right){\text{k}}}}+\sum\nolimits_{{\text{k}}=0}^{3}{{\text{w}}}_{{{\text{p}}}_{{\text{jkn}}}} {{\text{p}}}_{{{\text{p}}}_{{\text{ik}}}}+{{\text{e}}}_{{{\text{p}}}_{{\text{in}}}},$$

with ${{\text{y}}}_{{{\text{f}}}_{{\text{i}}}}$ and ${{\text{y}}}_{{{\text{p}}}_{{\text{in}}}}$ being the fertility and the ${\text{n}}$-th production phenotypes of cow ${\text{i}}$, ${\varvec{\upbeta}}_{\mathbf{f}}$ and ${\varvec{\upbeta}}_{\mathbf{p}}$ are the vectors of the fixed effects and ${\mathbf{x}}_{{\mathbf{f}}_{\mathbf{i}}}$ and ${\mathbf{x}}_{{\mathbf{p}}_{\mathbf{i}\mathbf{n}}}$ their incidence vectors, ${{\text{a}}}_{{{\text{f}}}_{{\text{s}}\left({\text{i}}\right){\text{k}}}}$ and ${{\text{a}}}_{{{\text{p}}}_{{\text{s}}\left({\text{i}}\right){\text{k}}}}$ the ${\text{k}}$-th genetic component of sire ${\text{s}}\left({\text{i}}\right)$, ${{\text{p}}}_{{{\text{p}}}_{{\text{ik}}}}$ the ${\text{k}}$-th animal component of the cow ${\text{i}}$ (including the permanent environment effect and a part of the genetic effect) for production, ${{\text{e}}}_{{{\text{f}}}_{{\text{i}}}}$ and ${{\text{e}}}_{{{\text{p}}}_{{\text{in}}}}$ the residuals, ${{\text{z}}}_{{{\text{f}}}_{{\text{jk}}}}$ the ${\text{k}}$-th Legendre polynomial of standardized THIf ${\text{j}}$, ${{\text{z}}}_{{{\text{p}}}_{{\text{jkn}}}}$ and ${{\text{w}}}_{{{\text{p}}}_{{\text{jkn}}}}$ the ${\text{k}}$-th Legendre polynomials of standardized THIp ${\text{j}}$. Note that ${{\text{z}}}_{{{\text{p}}}_{{\text{jkn}}}}$ and ${{\text{w}}}_{{{\text{p}}}_{{\text{jkn}}}}$ are equal for animals with phenotypes.

For CR, the vector of fixed effects ${\varvec{\upbeta}}_{\mathbf{f}}$ included: (i) herd-year as the contemporary group (261,087 levels in HOL; 66,544 levels in MON), (ii) month-year, (iii) age at insemination, in months (4 levels), (iv) interval between calving and insemination (3 levels), (v) sexed semen nested by year (30 levels), and day of the week (7 levels). The additive genetic effect ${{\text{a}}}_{{{\text{f}}}_{{\text{s}}\left({\text{i}}\right){\text{k}}}}$ was regressed using three functions: an intercept independent of THI and two THI-dependent functions (linear and quadratic functions). The vector ${\mathbf{z}}_{\mathbf{f}}$ contains three non-zero terms, equal to a constant and the first- and second-order Legendre polynomials of standardized THIf, respectively, ${\mathbf{a}}_{{\mathbf{f}}_{\mathbf{s}(\mathbf{i})}}$ the corresponding vector of additive sire regression coefficients, contains three values per animal. Preliminary analysis indicated that the effect of the service bull used for mating was low (< 1% variance) and this effect was not accounted for in our analyses. For PY, the vector of fixed effects ${\varvec{\upbeta}}_{\mathbf{p}}$ included: (i) herd-test-day as the contemporary group (1,572,611 levels in Holstein; 337,550 levels in Montbeliarde), (ii) days in milk defined in 10-d classes (12 levels), (iii) age at calving (20 levels), and (iv) days carried calf (days from successful insemination until test-day, converted into months, 5 levels). The additive sire effect ${{\text{a}}}_{{{\text{p}}}_{{\text{s}}\left({\text{i}}\right){\text{k}}}}$ and the permanent environmental effect of the cow ${{\text{p}}}_{{{\text{p}}}_{{\text{ik}}}}$ were each regressed using four functions: an intercept independent of THI and three THI-dependent functions (linear, quadratic, and cubic functions). The vectors ${\mathbf{z}}_{\mathbf{p}}$ and ${\mathbf{w}}_{\mathbf{p}}$ contain four non-zero terms on each line, equal to a constant and the first-, second-, and third-order Legendre polynomials of standardized THIp, respectively. ${\mathbf{a}}_{{\mathbf{p}}_{\mathbf{s}\left(\mathbf{i}\right)}}$ and ${\mathbf{p}}_{{\mathbf{p}}_{\mathbf{i}}}$, the corresponding vector of additive sire regression coefficients and vector of permanent environmental regression coefficients contain each four values per animal (i.e., per sire ${\text{s}}\left({\text{i}}\right)$ for ${\mathbf{a}}_{{\mathbf{p}}_{\mathbf{s}\left(\mathbf{i}\right)}}$ and per cow ${\text{i}}$ for ${\mathbf{p}}_{{\mathbf{p}}_{\mathbf{i}}}$). The arguments of the polynomials were standardized values of THI in the interval − 1 to + 1. For THIp, the raw range extended from 13 to 79 in Holstein and 11 to 79 in Montbeliarde, with zero corresponding to THI 46 in Holstein and THI 45 in Montbeliarde. Raw THIf values ranged from 14 to 77 in Holstein and 12 to 77 in Montbeliarde, with zero corresponding to THI 45 in Holstein and THI 44 in Montbeliarde. For both traits, the residuals ${{\text{e}}}_{{{\text{f}}}_{{\text{i}}}}$ and ${{\text{e}}}_{{{\text{p}}}_{{\text{in}}}}$ are considered heterogeneous across five THI classes (≤ 29, 30–39, 40–49, 50–59, ≥ 60). These classes were defined based on preliminary analyses and we chose to keep evenly spaced intervals although the differences were small between some adjacent classes.

The sire random regression variance matrix ${\text{var}}(\mathbf{a})=\mathbf{S}$ is a 7 × 7 symmetric matrix:

$$\mathbf{S}=\left[\begin{array}{cc}{\mathbf{S}}_{\mathbf{p}}& {\mathbf{S}}_{\mathbf{p}\mathbf{f}}\\ {\mathbf{S}}_{\mathbf{f}\mathbf{p}}& {\mathbf{S}}_{\mathbf{f}}\end{array}\right],$$

wit﻿h $${\mathbf{S}}_{\mathbf{p}}=\left[\begin{array}{c}{\upsigma }_{{{{\text{s}}}_{{\text{p}}}}_{0}}^{2}\\ {{\upsigma }_{{\text{s}}}}_{{{\text{p}}}_{1}{{\text{p}}}_{0}}\\ {{\upsigma }_{{\text{s}}}}_{{{\text{p}}}_{2}{{\text{p}}}_{0}}\\ {{\upsigma }_{{\text{s}}}}_{{{\text{p}}}_{3}{{\text{p}}}_{0}}\end{array} \begin{array}{c}{{\upsigma }_{{\text{s}}}}_{{{\text{p}}}_{0}{{\text{p}}}_{1}}\\ {\upsigma }_{{{{\text{s}}}_{{\text{p}}}}_{1}}^{2}\\ {{\upsigma }_{{\text{s}}}}_{{{\text{p}}}_{2}{{\text{p}}}_{1}}\\ {{\upsigma }_{{\text{s}}}}_{{{\text{p}}}_{3}{{\text{p}}}_{1}}\end{array} \begin{array}{c}{{\upsigma }_{{\text{s}}}}_{{{\text{p}}}_{0}{{\text{p}}}_{2}}\\ {{\upsigma }_{{\text{s}}}}_{{{\text{p}}}_{1}{{\text{p}}}_{2}}\\ {\upsigma }_{{{{\text{s}}}_{{\text{p}}}}_{2}}^{2}\\ {{\upsigma }_{{\text{s}}}}_{{{\text{p}}}_{3}{{\text{p}}}_{2}}\end{array} \begin{array}{c}{{\upsigma }_{{\text{s}}}}_{{{\text{p}}}_{0}{{\text{p}}}_{3}}\\ {{\upsigma }_{{\text{s}}}}_{{{\text{p}}}_{1}{{\text{p}}}_{3}}\\ {{\upsigma }_{{\text{s}}}}_{{{\text{p}}}_{2}{{\text{p}}}_{3}}\\ {\upsigma }_{{{{\text{s}}}_{{\text{p}}}}_{3}}^{2}\end{array}\right],$$

$${\mathbf{S}}_{\mathbf{f}}=\left[\begin{array}{c}{\upsigma }_{{{{\text{s}}}_{{\text{f}}}}_{0}}^{2}\\ {{\upsigma }_{{\text{s}}}}_{{{\text{f}}}_{1}{{\text{f}}}_{0}}\\ {{\upsigma }_{{\text{s}}}}_{{{\text{f}}}_{2}{{\text{f}}}_{0}}\end{array}\begin{array}{c}{{\upsigma }_{{\text{s}}}}_{{{\text{f}}}_{0}{{\text{f}}}_{1}}\\ {\upsigma }_{{{{\text{s}}}_{{\text{f}}}}_{1}}^{2}\\ {{\upsigma }_{{\text{s}}}}_{{{\text{f}}}_{2}{{\text{f}}}_{1}}\end{array}\begin{array}{c}{{\upsigma }_{{\text{s}}}}_{{{\text{f}}}_{0}{{\text{f}}}_{2}}\\ {{\upsigma }_{{\text{s}}}}_{{{\text{f}}}_{1}{{\text{f}}}_{2}}\\ {\upsigma }_{{{{\text{s}}}_{{\text{f}}}}_{2}}^{2}\end{array}\right],$$

$${\mathbf{S}}_{\mathbf{p}\mathbf{f}}=\left[\begin{array}{c}{{\upsigma }_{{\text{s}}}}_{{{\text{p}}}_{0}{{\text{f}}}_{0}}\\ {{\upsigma }_{{\text{s}}}}_{{{\text{p}}}_{1}{{\text{f}}}_{0}}\\ {{\upsigma }_{{\text{s}}}}_{{{\text{p}}}_{2}{{\text{f}}}_{0}}\\ {{\upsigma }_{{\text{s}}}}_{{{\text{p}}}_{3}{{\text{f}}}_{0}}\end{array} \begin{array}{c}{{\upsigma }_{{\text{s}}}}_{{{\text{p}}}_{0}{{\text{f}}}_{1}}\\ {{\upsigma }_{{\text{s}}}}_{{{\text{p}}}_{1}{{\text{f}}}_{1}}\\ {{\upsigma }_{{\text{s}}}}_{{{\text{p}}}_{2}{{\text{f}}}_{1}}\\ {{\upsigma }_{{\text{s}}}}_{{{\text{p}}}_{3}{{\text{f}}}_{1}}\end{array}\begin{array}{c}{{\upsigma }_{{\text{s}}}}_{{{\text{p}}}_{0}{{\text{f}}}_{2}}\\ {{\upsigma }_{{\text{s}}}}_{{{\text{p}}}_{1}{{\text{f}}}_{2}}\\ { {\upsigma }_{{\text{s}}}}_{{{\text{p}}}_{2}{{\text{f}}}_{2}}\\ {{\upsigma }_{{\text{s}}}}_{{{\text{p}}}_{3}{{\text{f}}}_{2}}\end{array}\right],$$

where ${\upsigma }_{{{{\text{s}}}_{{\text{p}}}}_{{\text{i}}}}^{2}$ is the variance of the ${\text{i}}$-th regression coefficient of the Legendre polynomial for PY, ${\upsigma }_{{{{\text{s}}}_{{\text{f}}}}_{{\text{i}}}}^{2}$ is the variance of the ${\text{i}}$-th regression coefficient of the Legendre polynomial for CR, ${{\upsigma }_{{\text{s}}}}_{{{\text{p}}}_{{\text{i}}}{{\text{p}}}_{{\text{j}}}}$ is the covariance between coefficients ${\text{i}}$ and ${\text{j}}$ of the regression for PY, ${{\upsigma }_{{\text{s}}}}_{{{\text{f}}}_{{\text{i}}}{{\text{f}}}_{{\text{j}}}}$ is the covariance between coefficients ${\text{i}}$ and ${\text{j}}$ of the regression for CR, and ${{\upsigma }_{{\text{s}}}}_{{{\text{p}}}_{{\text{i}}}{{\text{f}}}_{{\text{j}}}}$ is the covariance between the coefficient ${\text{i}}$ of the regression for PY and the coefficient ${\text{j}}$ of the regression for CR.

The additive sire variances and covariances at each THIp/f were estimated by pre- and post-multiplying the sire variance matrix $\mathbf{S}$ by the corresponding THI-coefficients of the Legendre polynomials using the formulas:

3 $${{\text{varS}}}_{{{\text{PY}}}_{{{\text{thi}}}_{{\text{p}}1}}}={\mathbf{z}}_{{{\text{p}}}_{{{\text{thi}}}_{{\text{p}}1}}}{\mathbf{S}}_{\mathbf{p}}{ \mathbf{z}}_{{{\text{p}}}_{{{\text{thi}}}_{{\text{p}}1}} ,}^{\mathrm{{\prime}}}$$

4 $${{\text{varS}}}_{{{\text{CR}}}_{{{\text{thi}}}_{{\text{f}}1}}}={\mathbf{z}}_{{{\text{f}}}_{{{\text{thi}}}_{{\text{f}}1}}}{\mathbf{S}}_{\mathbf{f}}{ \mathbf{z}}_{{{\text{f}}}_{{{\text{thi}}}_{{\text{f}}1}}}^{\mathrm{{\prime}}},$$

5 $${{\text{covS}}}{\left({{{\text{PY}}}_{{\text{thi}}}}_{{\text{p}}1}; {{{\text{PY}}}_{{\text{thi}}}}_{{\text{p}}2}\right)}={\mathbf{z}}_{{{\text{p}}}_{{{\text{thi}}}_{{\text{p}}1}}}{\mathbf{S}}_{\mathbf{p}}{ \mathbf{z}}_{{{\text{p}}}_{{{\text{thi}}}_{{\text{p}}2}} }^{\mathrm{{\prime}}},$$

6 $${{\text{covS}}}{\left({{{\text{CR}}}_{{\text{thi}}}}_{{\text{f}}1}; {{{\text{CR}}}_{{\text{thi}}}}_{{\text{f}}2}\right)}={\mathbf{z}}_{{{\text{f}}}_{{{\text{thi}}}_{{\text{f}}1}}}{\mathbf{S}}_{\mathbf{f}}{ \mathbf{z}}_{{{\text{f}}}_{{{\text{thi}}}_{{\text{f}}2}} }^{\mathrm{{\prime}}},$$

7 $${{\text{covS}}}{\left({{{\text{PY}}}_{{\text{thi}}}}_{{\text{p}}1}; {{{\text{CR}}}_{{\text{thi}}}}_{{\text{f}}2}\right)}={\mathbf{z}}_{{{\text{p}}}_{{{\text{thi}}}_{{\text{p}}1}}}{\mathbf{S}}_{\mathbf{p}\mathbf{f}}{ \mathbf{z}}_{{{\text{f}}}_{{{\text{thi}}}_{{\text{f}}2}} }^{\mathrm{{\prime}}}.$$

The additive genetic variances ${{\text{varG}}}_{{{\text{PY}}}_{{{\text{thi}}}_{{\text{p}}1}}}$ and ${{\text{varG}}}_{{{\text{CR}}}_{{{\text{thi}}}_{{\text{f}}1}}}$ and covariances at each THIp/f, ${{\text{covG}}}{\left({{{\text{PY}}}_{{\text{thi}}}}_{{\text{p}}1}; {{{\text{PY}}}_{{\text{thi}}}}_{{\text{p}}2}\right)}$, ${{\text{covG}}}{\left({{{\text{CR}}}_{{\text{thi}}}}_{{\text{f}}1}; {{{\text{CR}}}_{{\text{thi}}}}_{{\text{f}}2}\right)}$, and ${{\text{covG}}}{\left({{{\text{PY}}}_{{\text{thi}}}}_{{\text{p}}1}; {{{\text{CR}}}_{{\text{thi}}}}_{{\text{f}}1}\right)}$, were estimated by multiplying the corresponding sire additive variances and covariances by 4. With ${{\text{varG}}}_{{{\text{PY}}}_{{{\text{thi}}}_{{\text{p}}1}}}$, the genetic variance of PY was evaluated at THIp_1_; with ${{\text{varG}}}_{{{\text{CR}}}_{{{\text{thi}}}_{{\text{f}}1}}}$, the genetic variance of CR was evaluated at THIf_1_; ${{\text{covG}}}{\left({{{\text{PY}}}_{{\text{thi}}}}_{{\text{p}}1};{{\mathrm{ PY}}_{{\text{thi}}}}_{{\text{p}}2}\right)}$ was the genetic covariance between PY at THIp_1_ and PY at THIp_2_;  ${{\text{covG}}}{\left({{{\text{CR}}}_{{\text{thi}}}}_{{\text{f}}1}; {{{\text{CR}}}_{{\text{thi}}}}_{{\text{f}}2}\right)}$ was the genetic covariance between CR at THIf_1_ and CR at THIf_2_; and ${{\text{covG}}}{\left({{{\text{PY}}}_{{\text{thi}}}}_{{\text{p}}1}; {{{\text{CR}}}_{{\text{thi}}}}_{{\text{f}}2}\right)}$ was the genetic covariance between PY at THIp_1_ and CR at THIf_2_. ${\mathbf{z}}_{{{\text{p}}}_{{{\text{thi}}}_{{\text{p}}1}}}$ is the vector of covariates estimated at THIp_1_ and ${\mathbf{z}}_{{{\text{f}}}_{{{\text{thi}}}_{{\text{f}}1}}}$ is the vector of covariates estimated at THIf_1_.

The permanent environmental random regression variance matrix $\mathbf{P}$ and the permanent environmental variances, defined only for PY, were defined similarly as follows:

8 $${{\text{varP}}}_{{{\text{p}}}_{{{\text{thi}}}_{{\text{p}}1}}}={\mathbf{z}}_{{{\text{p}}}_{{{\text{thi}}}_{{\text{p}}1}}}\mathbf{P}{ \mathbf{z}}_{{{\text{p}}}_{{{\text{thi}}}_{{\text{p}}1}} ,}^{\mathrm{{\prime}}}$$

with $\mathbf{P}$ being the 4 × 4 symmetric permanent environmental random regression variance matrix.

Finally,${{\text{varR}}}_{{{\text{PY}}}_{{{\text{thi}}}_{{\text{p}}1}}}$,${{\text{varR}}}_{{{\text{CR}}}_{{{\text{thi}}}_{{\text{f}}2}}}$, and${{\text{covR}}}{\left({{{\text{PY}}}_{{\text{thi}}}}_{{\text{p}}1};{{\mathrm{ CR}}_{{\text{thi}}}}_{{\text{f}}2}\right)}$ are the residual variances and covariance at THIp_1_ and THIf_2_, respectively.

All these variances allowed calculation of the heritabilities at each THIp_i_ or THIf_j_ as:

9 $${{\text{h}}}_{{{\text{PY}}}_{{{\text{thi}}}_{{\text{pi}}}}}^{2}=\frac{4*{{\text{varS}}}_{{{\text{PY}}}_{{{\text{thi}}}_{{\text{pi}}}}}}{{{\text{varS}}}_{{{\text{PY}}}_{{{\text{thi}}}_{{\text{pi}}}}}+ {{\text{varP}}}_{{{\text{PY}}}_{{{\text{thi}}}_{{\text{pi}}}}}+ {{\text{varR}}}_{{{\text{PY}}}_{{{\text{thi}}}_{{\text{pi}}}}}},$$

10 $${{\text{h}}}_{{{\text{CR}}}_{{{\text{thi}}}_{{\text{fj}}}}}^{2}=\frac{4*{{\text{varS}}}_{{{\text{CR}}}_{{{\text{thi}}}_{{\text{fj}}}}}}{{{\text{varS}}}_{{{\text{CR}}}_{{{\text{thi}}}_{{\text{fj}}}}}+ {{\text{varR}}}_{{{\text{CR}}}_{{{\text{thi}}}_{{\text{fj}}}}}},$$

and the correlations between traits at each THIp_i_ and THIf_j_ as:

11 $${{{\text{r}}}_{{\text{g}}}}{\left({{\text{PY}}}_{{{\text{thi}}}_{{\text{pi}}}} ;{{\text{CR}}}_{{{\text{thi}}}_{{\text{fj}}}}\right)}=\frac{{{\text{covG}}}{\left({{{\text{PY}}}_{{\text{thi}}}}_{{\text{pi}}}; {{{\text{CR}}}_{{\text{thi}}}}_{{\text{fj}}}\right)}}{\sqrt{{{\text{varG}}}_{{{\text{PY}}}_{{{\text{thi}}}_{{\text{pi}}}}}*{{\text{varG}}}_{{{\text{CR}}}_{{{\text{thi}}}_{{\text{fj}}}}}}},$$

where ${{{\text{r}}}_{{\text{g}}}}{\left({{\text{PY}}}_{{{\text{thi}}}_{{\text{pi}}}};{\mathrm{ CR}}_{{{\text{thi}}}_{{\text{fj}}}}\right)}$ is the genetic correlation between PY at THIp_i_ and CR at THIf_j_.

The genetic correlation between the PY breeding value at THIp_i_ and the PY slope of the regression on (standardized) THIp_j_ was calculated as follows:

12 $${{{\text{r}}}_{{\text{g}}}}{\left({{\text{PY}}}_{{{\text{thi}}}_{{\text{pi}}}}; {{\text{slopePY}}}_{{{\text{thi}}}_{{\text{pj}}}}\right)}=\frac{{{\text{covG}}}{\left({{\text{PY}}}_{{{\text{thi}}}_{{\text{pi}}}}; {{\text{slopePY}}}_{{{\text{thi}}}_{{\text{pj}}}}\right)}}{\sqrt{{{\text{varG}}}_{{{\text{PY}}}_{{{\text{thi}}}_{{\text{pi}}}}}*{{\text{varG}}}_{{{\text{slopePY}}}_{{{\text{thi}}}_{{\text{pj}}}}}}},$$

with the variance of the PY slope at THIp_j_ computed using the derivative formula:

13 $${{\text{varG}}}_{{{\text{slopePY}}}_{{{\text{thi}}}_{{\text{pj}}}}}=4*\frac{{\mathbf{d}\mathbf{z}}_{{{\text{p}}}_{{{\text{thi}}}_{{\text{pj}}}}}}{{{\text{dTHI}}}_{{\text{p}}}} {\mathbf{S}}_{\mathbf{p}} {\left(\frac{{\mathbf{d}\mathbf{z}}_{{{\text{p}}}_{{{\text{thi}}}_{{\text{pj}}}}}}{{{\text{dTHI}}}_{{\text{p}}}}\right)}^{\mathrm{{\prime}}},$$

and the additive genetic covariance between the PY breeding value at THIp_i_ and the PY slope at THIp_j_ computed as:

14 $${{\text{covG}}}{\left({{\text{PY}}}_{{{\text{thi}}}_{{\text{pi}}}}; {{\text{slopePY}}}_{{{\text{thi}}}_{{\text{pj}}}}\right)}=4*{\mathbf{z}}_{{{\text{p}}}_{{{\text{thi}}}_{{\text{pi}}}}}{\mathbf{S}}_{\mathbf{p}}{ \left(\frac{{\mathbf{d}\mathbf{z}}_{{{\text{p}}}_{{{\text{thi}}}_{{\text{pj}}}}}}{{{\text{dTHI}}}_{{\text{p}}}}\right)}^{\mathrm{{\prime}}}.$$

The same formula was used to estimate the genetic correlation between the CR breeding value and the CR slope at two different values of THIf by adapting the matrix $\mathbf{S}$, the vector $\mathbf{z}$, and the derivative with regard to dTHIf.

Between traits, the correlation between the PY breeding value at THIp_i_ and the CR slope of the regression at THIf_j_ was calculated as:

15 $${{{\text{r}}}_{{\text{g}}}}{\left({{\text{PY}}}_{{{\text{thi}}}_{{\text{pi}}}} ;{{\text{slopeCR}}}_{{{\text{thi}}}_{{\text{fj}}}}\right)}=\frac{{{\text{covG}}}{\left({{\text{PY}}}_{{{\text{thi}}}_{{\text{pi}}}} ;{\mathrm{ slopeCR}}_{{{\text{thi}}}_{{\text{fj}}}}\right)}}{\sqrt{{{\text{varG}}}_{{{\text{PY}}}_{{{\text{thi}}}_{{\text{pi}}}}}* {{\text{varG}}}_{{{\text{slopeCR}}}_{{\text{fj}}}}}}.$$

The approximate standard errors of the variance components were derived from the average information matrix as proposed in [25] and the standard errors of the genetic correlations were approximated as proposed in [26].

All fixed effects and variance components were estimated using the WOMBAT software [27].

In preliminary analyses, different orders of Legendre polynomials were tested in the MON breed. For each trait, models with different orders of Legendre polynomials (order two and three, featuring three and four polynomial coefficients, respectively) were compared using the Akaike information (AIC) and the Bayesian information criteria (BIC). The results with respect to these criteria of model assessment indicated that the best model was that of a third order polynomial for PY (cubic, with four polynomial coefficients—for order 0, 1, 2, and 3), and that of a second order polynomial (quadratic, with three polynomial coefficients—for order 0, 1, and 2) for CR (Table 2).

**Table 2:** Akaike information criterion (AIC) and Bayesian information criteria (BIC) for all tested models in Montbeliarde

	Number of polynomials considered for each trait		AIC	BIC
	Protein yield	Conception rate		
Model 1	4	4	21,864,428.1	21,865,475.8
Model 2	4	3	21,864,415.6	21,865,348.4
Model 3	3	3	21,864,934.0	21,865,713.5
Model 4	3	4	21,865,198.8	21,866,118.8

## Results

### Genotype-by-THI interactions within traits

**Fig. 3:**
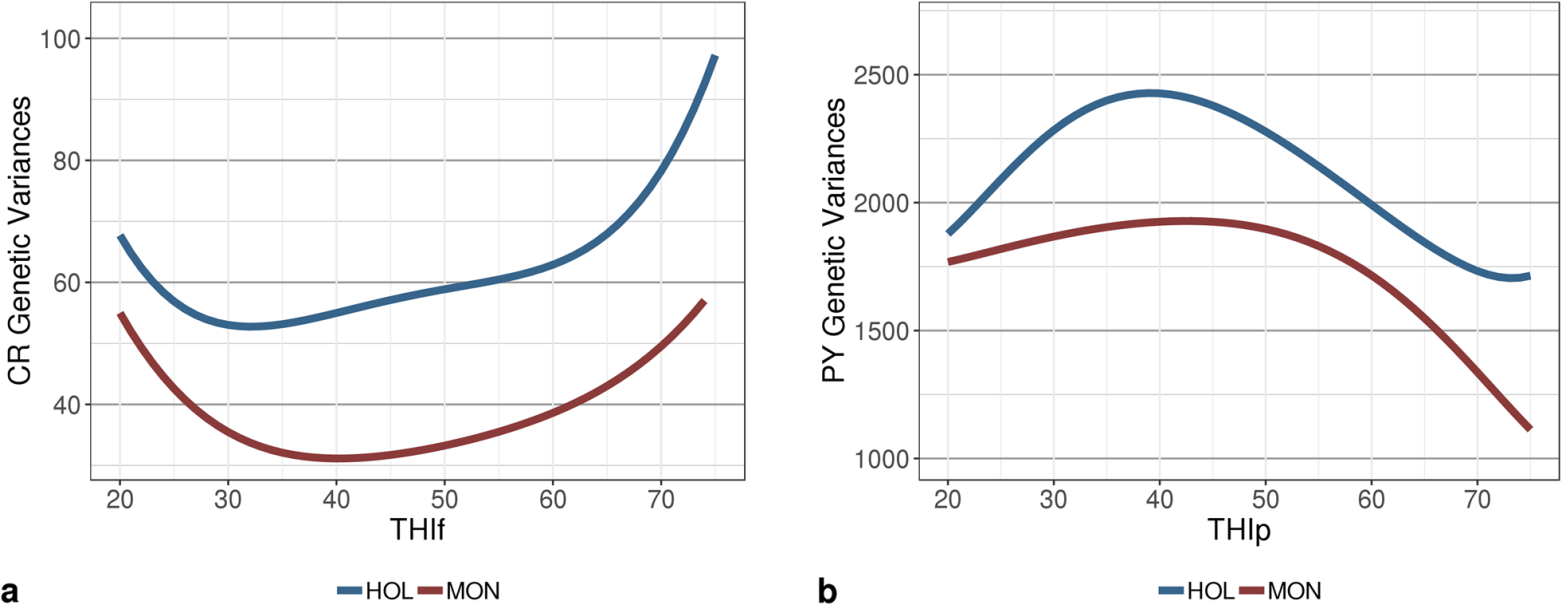
Trajectories of estimates of additive genetic variance for **a** conception rate (CR) and **b** protein yield (PY) with changing temperature-humidity index (THI) in Holstein (HOL) and Montbeliarde (MON) breeds. *THIp* THI for protein yield, *THIf* THI for conception rate

**Fig. 4:**
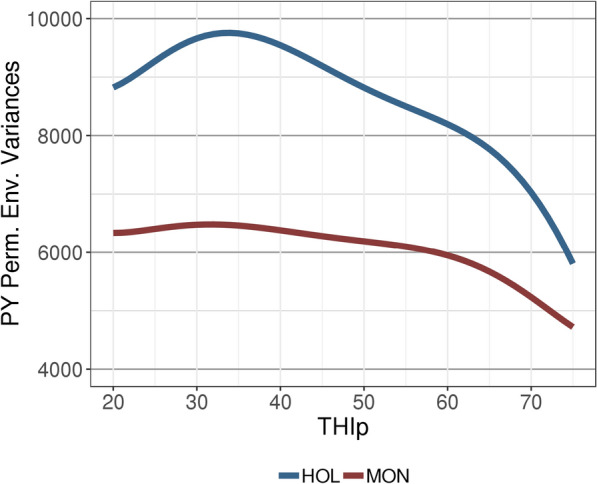
Trajectories of estimates of permanent environmental variances for protein yield (PY) with changing temperature-humidity index (THI) in Holstein (HOL) and Montbeliarde (MON) cows. *THIp* THI for protein yield, *THIf*  THI for conception rate

**Fig. 5:**
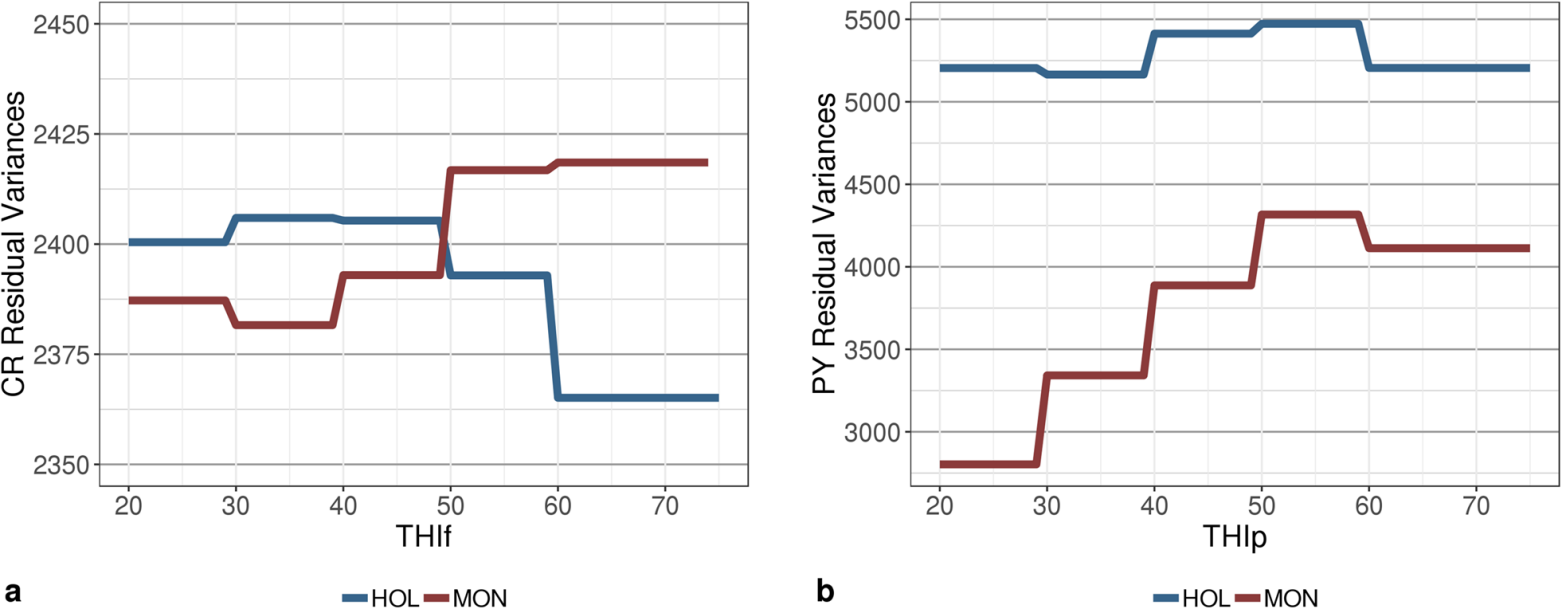
Trajectories of estimates of residual variance for **a** conception rate (CR) and **b** protein yield (PY) with changing temperature-humidity index (THI) in Holstein (HOL) and Montbeliarde (MON) cows. *THIp* THI for protein yield, *THIf*  THI for conception rate

The trajectories of the estimated genetic and permanent environmental variances followed the same trend in both breeds; however, the estimated values were higher for HOL than for MON (Figs. 3 and 4). For CR, the largest additive genetic variances were observed for extreme values of THI (i.e., THIf < 30 and THIf > 60), but for PY, these extreme THI values were associated with the lowest estimates of additive genetic variance (Fig. 3). Similar to the trends for genetic variance, the permanent environmental variance for PY tended to decrease with extreme THIp values for both breeds, but this pattern was more pronounced for HOL (Fig. 4). For MON, the residual variances of both traits increased with THI, whereas for HOL the residual variance tended to decrease for CR at values of THI > 50, and for PY at THI > 60 (Fig. 5).

**Fig. 6:**
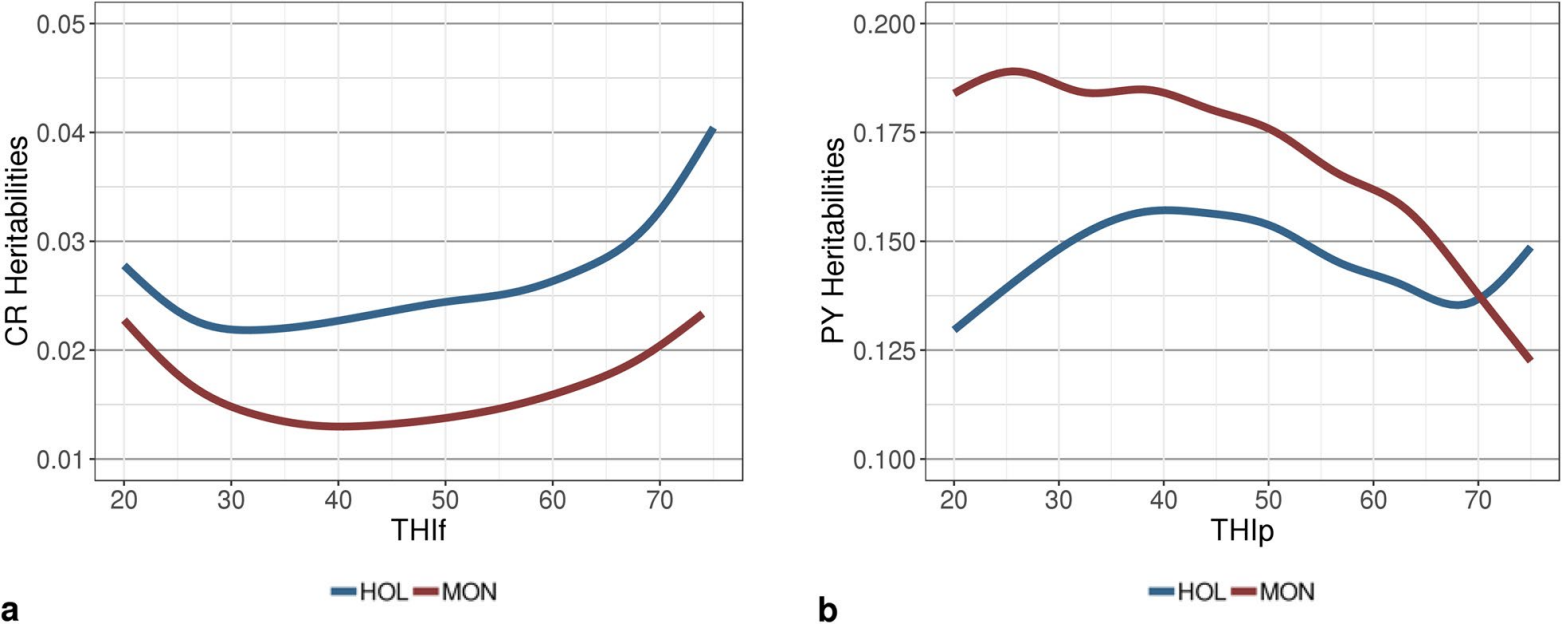
Smoothed trajectories of estimates of heritability for **a** conception rate (CR) and **b** protein yield (PY) with changing temperature-humidity index (THI) in Holstein (HOL) and Montbeliarde (MON) cows. *THIp* THI for protein yield, *THIf*  THI for conception rate

Figure 6 presents the trends in heritability as a function of THI. These reflected the patterns that were observed for the additive genetic variances, with a tendency for heritabilities to increase at extreme THIf values for CR, and conversely, to decrease at extreme THIp values for PY. In spite of the general declining trend for PY, a rebound was observed in HOL heritabilities (and genetic variances) at the highest THI values. Although additive genetic variances were larger for HOL than for MON, the heritabilities of PY for the former breed were lower than those for the latter due to the much larger estimates of residual and permanent environmental variances for HOL (Figs. 3, 4, 5 and 6).

**Fig. 7:**
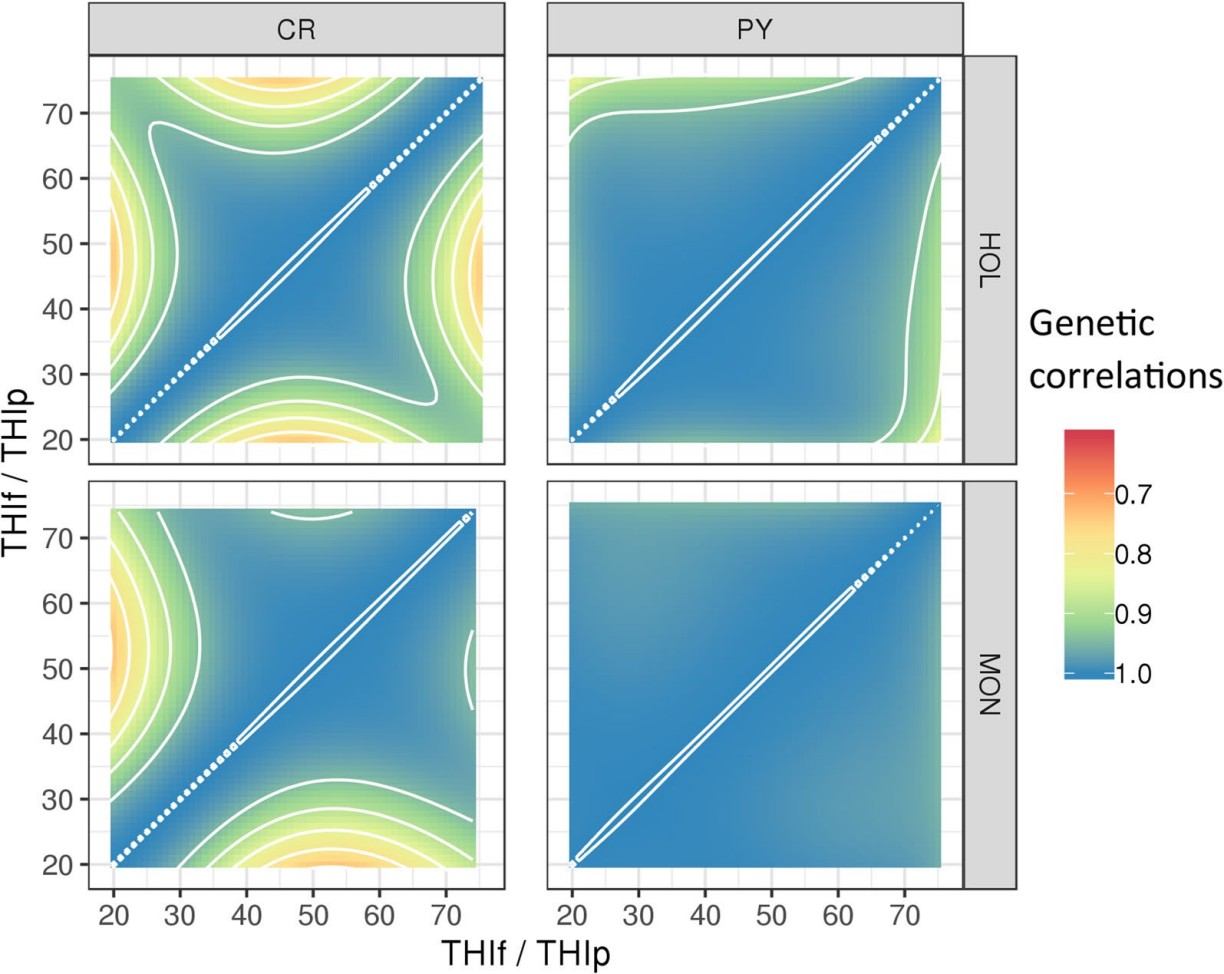
Estimates of within-trait genetic correlations at different values of temperature-humidity index (THI) for conception rate (CR, left-hand graphs) and protein yield (PY, right-hand graphs) in Holstein (HOL, top graphs) and Montbeliarde (MON, bottom graphs). White contour lines separate genetic correlation classes differing by 0.05. *THIp* THI for protein yield, *THIf * THI for conception rate

The genetic correlations within traits across THI values were lower for HOL than for MON, and lower for CR than for PY (Fig. 7). Nonetheless, interactions between genotype and THI remain limited, with correlations generally high and mostly above 0.80. In fact, the majority of the estimated genetic correlations within PY were higher than 0.85 and 0.95 for HOL and MON, respectively. For CR, these genetic correlations dropped to 0.75 for both breeds when calculated between extreme and intermediate THI values.

Recent work by our group found that, even when genetic correlations suggest weak genetic-by-THI interactions, some reranking of animals can occur at high THI values [8]. The same pattern was detected in the current study: some animals experienced a decrease in their estimated breeding values (BV) while others moved up in the ranking. These changes in the ranking of estimated BV can be quantified by calculating the slope at a given THI. The estimates of the genetic variances of the traits at intermediate THI (THI = 50) and at high THI (THI = 70), and the estimates of the genetic variances of the slope of the trait at THI 70 (i.e. the derivative at THI 70) are in Table 3. The estimates of the genetic standard deviations of the slopes at high THI (THI = 70) and the proportions of their variability out of the total genetic variability of each trait at THI = 70 are in Table 4. The genetic standard deviations of the slopes ranged from 0.19 to 0.35 points per unit of additional THI for CR and around 1 g/d per unit of additional THI for PY. These genetic standard deviations of the slopes accounted for less than 5% of the overall genetic standard deviation of the traits at THI 70, regardless of the breed. Although the magnitude of these effects was limited, they were estimated for only a short period of heat stress (Tables 3 and 4). In the future, heat waves are expected to be more frequent and more intense, making it likely that losses in CR and PY will accumulate. Therefore, we also investigated the genetic correlations of these slopes at high THI with the BV at intermediate THI, which enables the prediction of potential consequences from current selection decisions (based on BV expressed at intermediate THI) in a breeding scenario taking place in future forecasted climate conditions (higher THI). Table 5 presents the within-trait genetic correlations between BV at THI 50 and slope at THI 70. The genetic correlation for PY was negative for both breeds (−0.37 ± 0.06 for HOL and −0.71 ± 0.09 for MON), while for CR the trends differed between breeds. For MON, the BV for CR at THI 50 tended to be favorably correlated with the slope at THI 70, meaning that, for this breed, the most fertile animals under the current climate conditions likely remain the most fertile under future forecasted conditions (r_g_ =  0.37 ± 0.38). Instead, for the HOL breed, the genetic correlation was close to zero (r_g_ = −0.03 ± 0.09). This means that the current selection for the CR does not appear to have any consequence on the slope under a heat stress scenario.

**Table 3:** Genetic variances of the traits at temperature-humidity index (THI) 50 and THI 70, and genetic variances of the slopes at THI 70

	CR (%^2^)	PY (g^2^/d^2^)
Genetic variance of the trait at THI = 50		
HOL	58.9 (2.53)	2277 (56)
MON	33.6 (3.85)	1887 (87)
Genetic variance of the trait at THI = 70		
HOL	78.3 (5.68)	1733 (55)
MON	49.7 (8.95)	1322 (84)
Genetic variance of slope at THI = 70 (per unit of additional THI^2^)		
HOL	0.12 (0.01)	1.31 (0.03)
MON	0.03 (0.006)	0.99 (0.03)

Standard errors in brackets

**Table 4:** Genetic standard deviation (sd) of the trait and genetic sd of the slope at temperature-humidity index (THI) 70, and proportion of the genetic sd of the slope compared to the overall sd of the trait at THI70

	CR	PY
Genetic standard deviation of the trait at THI = 70		
HOL	8.8%	42 g/d
MON	7.1%	36 g/d
Genetic standard deviation of slope at THI = 70 (per unit of additional THI)		
HOL	0.35%	1.14 g/d
MON	0.19%	1.00 g/d
Ratio between genetic standard deviation of the slope at THI = 70 (per unit of additional THI) and genetic standard deviation of the trait at THI = 70 (%)		
HOL	4.0%	2.7%
MON	2.6%	2.7%

**Table 5:** Estimates of genetic correlations (standard errors in brackets) between breeding values (BV) and slopes at various temperature-humidity index (THI) combinations

	HOL	MON
*Genetic correlations within traits*		
r_g_ (BV_CR(THIf=50)_; slope_CR(THIf=70)_)	− 0.03 (0.09)	+ 0.37 (0.38)
r_g_ (BV_PY(THIp=50)_; slope_PY(THIp=70)_)	− 0.37 (0.06)	− 0.71 (0.09)
*Genetic correlations between traits*		
Genetic correlations between BV		
r_g_ (BV_CR(THIf=50)_; BV_PY(THIp=50)_)	− 0.14 (0.02)	− 0.03 (0.06)
r_g_ (BV_CR(THIf=70)_; BV_PY(THIp=70)_)	− 0.08 (0.04)	− 0.16 (0.08)
r_g_ (BV_CR(THIf=50)_; BV_PY(THIp=70)_)	− 0.06 (0.03)	+ 0.01 (0.06)
r_g_ (BV_CR(THIf=70)_; BV_PY(THIp=50)_)	− 0.18 (0.03)	− 0.20 (0.08)
Genetic correlations between BV and slope		
r_g_ (BV_PY(THIp=50)_; slope_CR(THIf=70)_)	− 0.15 (0.08)	− 0.54 (0.35)
r_g_ (BV_CR(THIf=50)_; slope_PY(THIp=70)_)	+ 0.33 (0.06)	+ 0.20 (0.13)
Genetic correlations between slopes		
r_g_ (slope_CR(THIf=70)_; slope_PY(THIp=70)_)	+ 0.24 (0.14)	+ 0.27 (0.45)

### Genotype-by-THI interactions between traits

**Fig. 8:**
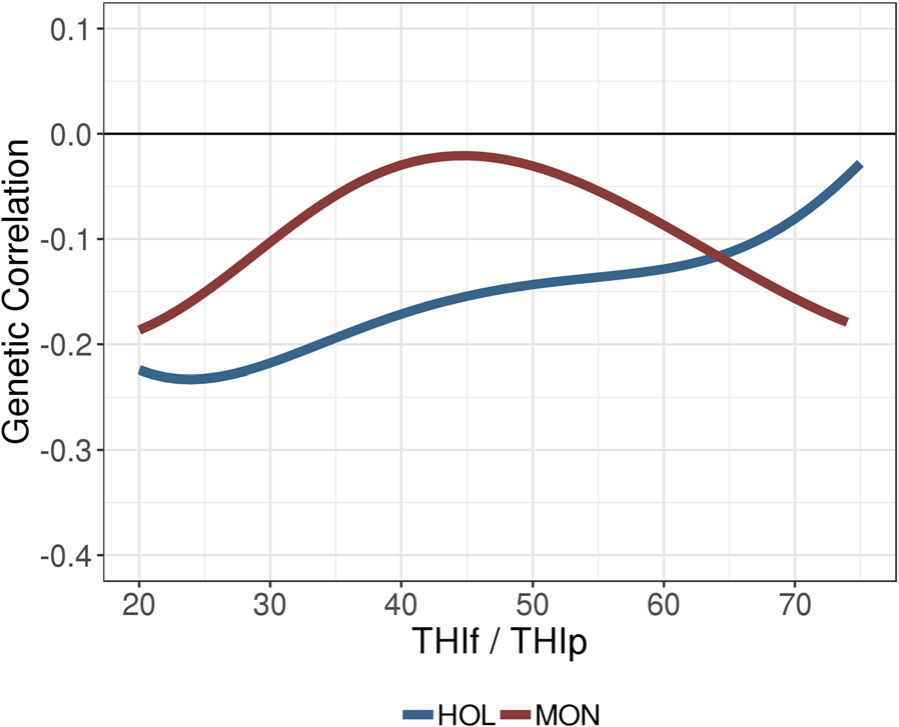
Estimates of genetic correlations between conception rate and protein yield at a given temperature-humidity index (THIf = THIp) in Holstein (HOL) and Montbeliarde (MON) cows

**Fig. 9:**
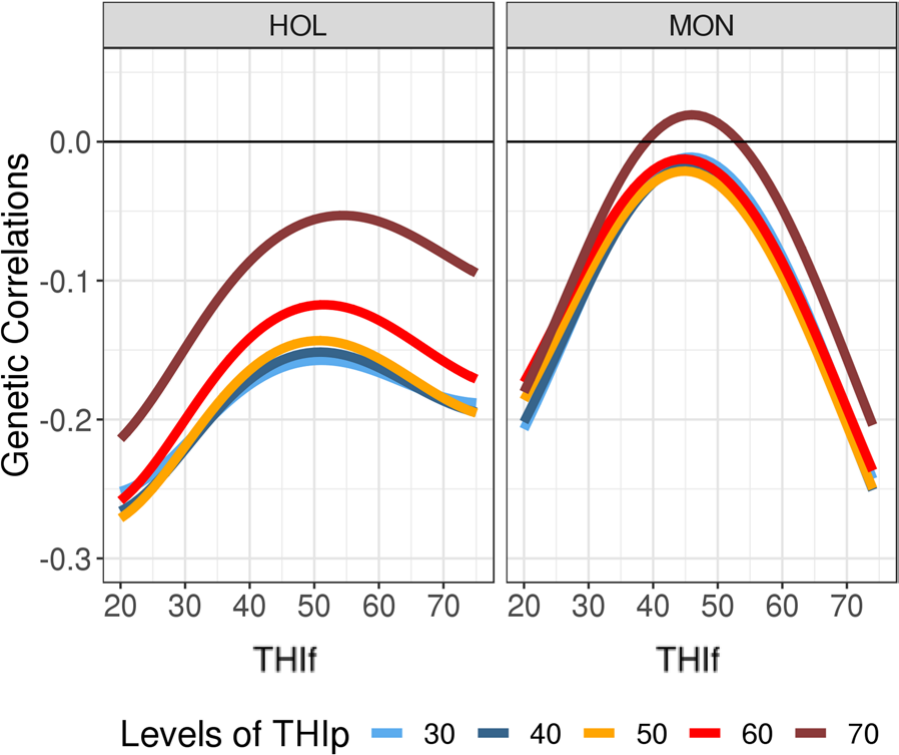
Estimates of genetic correlations between conception rate and protein yield with changing levels of temperature-humidity index for fertility (THIf) and for five levels of temperature-humidity index for production (THIp). Results are given for Holstein (HOL, left) and Montbeliarde (MON, right)

The genetic correlations between traits are presented in Figs. 8 and 9 and in Table 5. Unlike the additive genetic variances, the additive genetic covariances between traits -and thus the additive genetic correlations- evolved differently for the two breeds (Figs. 8 and 9). All genetic correlations between traits at a given THI (e.g., THIp = THIf) were low, ranging from 0 to −0.2. However, for HOL these increased with THI (Fig. 8), whereas for MON, estimates were close to zero at intermediate THI and mildly negative at extreme THI (e.g., −0.16 ± 0.08 at THIf = THIp = 70, Fig. 8), suggesting some deterioration of the production-fertility trade-off when THI is extreme. Figure 9 shows the trajectories of genetic correlations between CR and PY along the gradient of THIf and for five THIp values ranging from 30 to 70. For both breeds, the genetic correlations between CR and PY varied little regardless of the value of THIp considered for PY. For instance, the genetic correlations between CR at THIf 70 and PY at THIp 50 are close to the genetic correlations between CR at THIf 70 and PY at THIp 70 (−0.18 vs −0.08 for HOL, and −0.20 vs −0.16 for MON, Fig. 9 and Table 5).

Table 5 reports the genetic correlations between various combinations of breeding values and slopes of regression at different values of THI. The genetic correlation between breeding values at THI 50 and the slope at THI 70 represents the relationship between the average current breeding value and the trend of breeding values in warmer conditions. The correlation between PY level at THIp 50 and CR slope at THIf 70 was negative for both breeds, although more strongly for MON than for HOL (r_g_ = −0.54 ± 0.35 and −0.15 ± 0.08, respectively). These correlations indicated that the cows that are high-yielding under current climate conditions are expected to have a stronger decline in fertility under future forecasted climate conditions. Finally, the correlation between CR and PY slopes was positive at THI 70 for both breeds (r_g_ =  0.24 ± 0.14 for HOL and 0.27 ± 0.45 for MON), which suggests that the animals with the strongest favorable responses to THI were the same for both traits.

## Discussion

### Choice of the model and distribution of THI data

In order to achieve accurate estimates for genetic correlations at various values of THI for a low-heritability trait such as CR, we chose to use a very large dataset. However, this required a reduction in the complexity of the model to reduce the computational burden. Specifically, we removed genetic-by-DIM interactions by using only PY records from the middle of the lactation (80–200 DIM), which greatly simplified the model. This range of DIM presents highly correlated BV, with stable genetic variance [6, 23, 24]. However, due to the partial seasonality of calving, many first calvings occurred in fall. For these lactations, PY performances recorded in summer were not included because they were later than 200 days in milk. Consequently, the average THIp for MON was lower than that observed in a previous analysis of MON PY [8]. In the current study, the proportion of PY data obtained under hot conditions (i.e., with THIp ≥ 70) represented only 1.2% (HOL) and 1.4% (MON) of the total. In spite of this, the amount of information was large enough to obtain accurate estimates. As shown in Fig. 2, the distribution of THI values in this study covered a large range from low to high, and accurately reflects the climatic variability in France. However, farmers tend to avoid breeding during the warm season, and as a result, the majority of the first services are planned in fall and winter. In the original data before filtering, 61% (HOL) and 67% (MON) of primiparous first services were performed between October and March, meaning that only 0.7% (HOL) and 1.2% (MON) of the inseminations were recorded at THIf ≥ 70. Given this disparity, a very large dataset was needed to generate accurate estimates. Accordingly, to make model computation feasible, a sire model was used.

### Genetic-by-THI interactions within trait

#### Conception rate

Under intermediate climate conditions (THI from 30 to 60), estimates of the CR heritability were lower than 0.025. These values are in agreement with the existing literature on fertility traits in dairy cattle, which commonly reports heritabilities between 0.01 and 0.05 for the two breeds studied here [13, 21, 28–36]. In earlier studies of fertility in French dairy cattle populations, Boichard et al. [13, 28] estimated the heritability of successful postpartum insemination for HOL (0.013 to 0.022) and MON (0.011), and their values were close to our estimates at the intermediate THIf of 50 (0.024 for HOL and 0.014 for MON, Fig. 6). The estimates of additive genetic standard deviation (5 points) from that study were also similar to, although somewhat lower than, our results (7.7 points in HOL and 5.8 points in MON at THIf 50, Table 3). The estimates of CR heritability were higher at extreme values of THI, both high and low (up to 4% in HOL and 2.3% in MON). Generally, in studies on genetic-by-climate interactions with random regression models or with multivariate analyses of performance in various climate zones, a slight increase in the heritability of cow fertility traits is reported in warmer environments (e.g., calving interval [37]; conception rate at first service [21]; conception rate in second parity [40]; 25-d non-return rate at first service [38]; 90-d non-return rate at first service [34]). Brügemann et al. [21] highlighted that, for random regression models with Legendre polynomials, this increase in heritabilities at extreme THI is observed regardless of the production system and THI period considered (before, after, or on the day of insemination). This increase in heritability with extreme THI might be explained by an enhanced genetic differentiation of functional traits in harsher environments [21], a phenomenon that has also been reported for udder health traits [8].

Our results did not support the existence of strong genetic-by-THI interactions for CR (Fig. 7). Some genetic correlations lower than 0.80 were observed between extreme and intermediate THI values; however, extreme THI values (i.e., lower than 21 or higher than 76) were scarce in our datasets (Fig. 2). Because of this underrepresentation of extreme THI, we are unable to fully distinguish true genetic-by-environment interactions from border effects, a well-known drawback of random regression models that is accentuated when data are scarce at boundaries [23]. Results from the literature on genetic-by-climate interactions for fertility traits are contradictory. High genetic correlations (i.e., higher than 0.78) between THI conditions have been observed for the 25-d non-return rate at first service ([38], in Australian Holstein), days open ([39], in US Holstein), conception rate ([40], in Japanese Holstein), and calving interval ([37], in Iranian Holstein). However, results for Brazilian Holsteins revealed stronger interactions [41]. In that study, the average genetic correlation between the non-return rate at 56 days at opposite extremes of the THI scale (THI 61 and 76) reached -0.12. Similarly high genetic x THI interactions were also found in Brazilian Nellore heifers [42] for reproductive traits.

#### Protein yield

All values of PY variance estimated in this study for the MON breed with a sire model were in agreement with previous estimates obtained for the same breed with a single-trait animal model [8]. Most of the differences observed in the present study compared to previous research can be explained by the size of our dataset or possibly by our use of a sire model. As expected, genetic variances were slightly larger for HOL than for MON due to a higher production level and scale effect. In line with the results of Carabaño et al. [7], our heritability estimates tended to decrease with increasing temperature. However, overall, the results from the literature on variation in heritabilities for production traits are not completely consistent. In contrast to our findings, Aguilar et al. [43] and Ravagnolo and Misztal [44] highlighted an increase in the heritability of yields starting from THI 72. Here, a slight rebound in PY heritability and genetic variance was also observed starting from THI 70 for HOL only (Figs. 3 and 6), but due to the lack of data at THI ≥ 70, this minor increase is most likely due to border effects.

Our estimates of additive genetic correlations for PY remained high across values of THI (Fig. 7), which confirms previous results obtained in MON and is consistent with the findings of Brügemann et al. [6] and Cheruiyot et al. [45]. However, when comparing extreme mean temperatures such as –1 and 34 °C, Carabaño et al. [7] were able to detect much stronger interactions (r_g_ = 0.24). Although the temperature range in France is quite wide, a daily average temperature of 34 °C, with performance data recorded, is still rare. Based on the range of humidity usually observed in France at high temperatures, such an extreme situation would correspond to a THI between 80 and 89.

### Evolution of the trade-off between fertility and production with changing THI

In an intermediate-THI scenario (i.e., THI 50), the estimated genetic correlations between CR and PY were only mildly negative for HOL (−0.14 ± 0.02) and close to zero for MON (−0.03 ± 0.06, Fig. 8 and Table 5). These results are consistent with those of Kadarmideen et al. [32] and Sun et al. [46], who observed little or no genetic correlation between production and fertility. However, this lack of a trade-off between production and fertility is in conflict with what is generally found in high-producing dairy cows. Indeed, most studies have revealed an antagonism between CR or non-return rate and 305-d production, with consistent results among milk, fat, or protein yields. The magnitude of this antagonism varies widely among reports, however, with values ranging from weak (less than −0.2 [32, 47]) or moderate (between −0.2 and −0.4 [14, 36]) to strong (up to −0.5 [15, 31]). It is important to emphasize that the correlation with 305-d yields also reflects the depressive effect of gestation on persistency in the last third of lactation; the true antagonism between fertility and production must be measured earlier in the lactation. When genetic correlations are estimated between CR or non-return rate and test-day milk yields, the results tend to be more stable, with low to moderate genetic correlations between fertility and production (between −0.10 and −0.25 in [30, 35, 48]; up to −0.41 in [34]). In studies on French dairy cattle, Boichard et al. [13, 28] found a greater opposition between CR and PY (−0.25 to −0.36 for HOL and −0.35 for MON) than we did here, and an even greater opposition between CR and milk yield. Because their studies focused on production at the beginning of the lactation (up to 100 days in milk), they suggested that these correlations reflected the influence of the energy balance of the cow. In the present study, only mid-lactation performances were considered, between 80 and 200 days in milk. At this lactation stage, the energy balance is no longer negative and PY is free from gestation effects, and the genetic correlation between PY and CR is therefore less unfavorable than in the first 100 days of lactation.

Using THI-dependent bivariate random regression models, it is possible to estimate the genetic correlations between CR and PY for any combination of THIf and THIp, and thus to foresee the evolution of the correlation between these two traits in forecasted warmer conditions. Although the magnitude of this evolutionary change is not strong, its direction differs for the two breeds studied: an increase is predicted for HOL (up to around r_g_ = −0.08 ± 0.04 at THI 70, Fig. 8 and Table 5), whereas a slight decrease is predicted for MON (around r_g_ = −0.16 ± 0.08 at THI 70, Fig. 8 and Table 5). While the results presented to date indicated a similar amount of genetic determinism in HOL and MON, this finding may suggest a slight divergence between these two breeds. This relatively small change in the genetic correlation between PY and CR with changing THI was unexpected, because a stronger antagonism was anticipated by many dairy cattle specialists. To our knowledge, no similar studies have yet been conducted and our results cannot be compared with data from the literature.

An illustration of the potential consequences of selection on PY under current conditions for CR in future environmental conditions is given in Fig. 9. A THIp equal to 50 is approximately the average THI value under current conditions (Table 1) and a frequent situation encountered by French dairy cattle (Fig. 2). Thus, it can be considered as the average meteorological condition of the current selection regime in France. In both breeds, ongoing selection on PY was found to have a mildly negative effect on CR at THI 70. For MON, the current selection on PY may worsen the antagonism between the two traits in the future (r_g_ = −0.20 ± 0.08 vs −0.03 ± 0.06 today); instead, for HOL, the scenario would not be worse than at present. Indeed, the genetic correlation between PY at THIp 50 and CR at THIf 70 (r_g_ = −0.18 ± 0.03) was only slightly different from the current situation (r_g_ between PY at THIp 50 and CR at THIf 50 = −0.14 ± 0.02).

### Selection on heat tolerance

For CR in MON, the likely positive genetic correlation between the breeding value at intermediate THI and the slope at high THI (r_g_ = 0.37 ± 0.38, Table 5) suggests that cows that are already the poorest reproducers at THI 50 may experience even more difficulties under heat stress. The same phenomenon of amplified differences during heat stress was also observed for somatic cell score for MON [8]. However for HOL, this correlation is, instead, close to zero. For this breed, the expected evolution (either an increase or decrease) in the ranking of animals exposed to a heat stress scenario is not correlated with the ranking at intermediate THI. These results differ from those in the available literature, which generally report negative genetic correlations between breeding values under normal climate conditions (i.e., no heat stress) and slope at high THI in US [34, 49], Italian [50], and Iranian Holsteins [51]. These correlations ranged from −0.25 to −0.95 depending on the trait (45-d, 60-d, or 90-d non-return rates [34]); from −0.35 to −0.82 according to parity [49]; from −0.35 to −0.45 also according to the parity of the cow [50]; and from −0.18 to −0.47 depending on the trait (CR or 45-d and 90-d non-return rates [51]). However, all of these previous estimates were obtained using broken line models, which is not the case in our analyses. The broken line model assumes the presence of a neutral zone in which THI has no effect, i.e., an intercept; beyond this neutral zone, the slope, i.e., the tolerance effect, is present. In a preliminary analysis of CR, we fitted such a broken line model to our HOL data, and, although the broken line model emphasized the slope compared to the random regression model, the genetic correlations between intercept and slope remained close to zero. Ravagnolo and Misztal [34] observed slightly different results depending on whether this trait was evaluated with a single-trait model or combined with a production trait in a two-trait model. These authors hypothesized that analyses of the non-return rate without the correlated production trait resulted in biased estimates [34]. Here, although, in a preliminary analysis of CR using random regression on THI and a single-trait model, we obtained results that were very close to those obtained with the two-trait model with PY (results not shown). Overall, in our study, estimates of the variance components of the fertility trait were not substantially affected by either the choice of the random regression model or by the joint analysis of fertility and production. This may explain the discrepancies we observed between our results and those reported in the literature.

With respect to the genetic correlation between the BV of PY at THI 50 and the slope at THI 70, although the relationship was negative in both breeds, it was much stronger for MON than for HOL (−0.37 ± 0.06 for HOL and −0.71 ± 0.09 for MON, Table 5). These findings are consistent with those reported by Ravagnolo and Misztal [34] (r_g_ = −0.45) and provide additional support to previous results obtained by our group for MON [8]. In our earlier study on production traits (as well as somatic cell score), we discussed whether the slope at high THI was a good predictor of heat tolerance in cows. Specifically, our question was whether a negative slope for PY at high THI actually reflects a lower tolerance to heat stress, or whether it simply reflects a self-protective mechanism to prioritize other functions. The moderate, but positive, genetic correlation between the PY and CR slopes at THI 70 for HOL (r_g_ = 0.24 ± 0.14, Table 5) suggests that the slope of production traits at high THI could be an indicator of heat tolerance. Since production traits are more heritable and more intensively recorded than fertility traits, the inclusion of this slope for production traits (with or without that for CR) in breeding goals may be effective in improving overall heat tolerance in dairy cattle without compromising reproductive capacity at high temperatures. With their THI-dependent, two-trait, and broken line model, Ravagnolo and Misztal [34] estimated a genetic correlation close to zero between the two traits of heat tolerance (i.e., slopes for both 90-d non-return rate and milk yield), and they concluded that the metabolic and physiological processes that are responsible for heat tolerance are different for milk and reproduction traits. Our estimates of the genetic correlations between PY and CR slopes in a heat stress scenario (THI 70) were positive but still quite moderate. Therefore, selection for heat tolerance using the PY slope may not necessarily select for the same physiological processes as would selection based on the CR slope. Nevertheless, the results of this study suggest that these two heat tolerance traits are not antagonistic.

The use of THI-dependent bivariate random regression models can also provide information on what the consequences of the current selection program might be for PY and CR in future THI conditions. The performance of PY at THI 50 presented a negative genetic correlation with that of CR at THI 70, i.e., in the forecasted warmer future conditions (r_g_ = −0.2 for both breeds, Fig. 9 and Table 5). Likewise, the BV of PY at THI 50 were also negatively correlated with the slopes for CR at THI 70 (r_g_ = −0.15 ± 0.08 for HOL and −0.54 ± 0.35 for MON, Table 5). These genetic correlations suggest that the current selection for PY may have a negative impact on future CR, but probably not much more than in current conditions (especially for HOL). Conversely, the current selection regime for CR would have no effect on PY in the warmer conditions of the future (r_g_ = −0.06 for HOL and r_g_ = 0.01, for MON, Table 5), and a minor positive impact on the slope of PY in a heat stress scenario (r_g_ = 0.33 ± 0.06 for HOL and r_g_ = 0.20 ± 0.13 for MON, Table 5). Furthermore, due to the high values observed for the genetic correlations between the BV of PY across the gradient of THI (r_g_ > 0.95 for the two breeds, Fig. 7), it seems that the current selection for PY would be able to select for animals with high BV for PY over the whole THI gradient, including the THI values associated with a heat stress scenario. However, these animals will still be more sensitive to heat stress with respect to both PY and CR. Overall, our study indicated that both PY and CR are negatively affected by increasing values of THI, which highlights the importance of selecting for greater heat tolerance. Such efforts must be designed to take the evolution of both production and functional traits under heat stress conditions into account.

## Conclusions

The aim of this study was to predict the evolution of fertility in Holstein and Montbeliarde females and to identify relevant traits for selection in the context of climate change. Our results revealed that, for PY, genetic variance and heritability decreased with increasing THI; instead, the opposite pattern was observed for CR, indicating that adverse conditions are potentially favorable to the genetic expression of reproductive traits. When PY was measured in mid-lactation, i.e. when its genetic determinism is stable and cows are in a situation of neutral energy balance, its antagonism with CR was lower than usually assumed. Surprisingly, this genetic correlation remained more or less stable, between 0 and -0.2, in all THI conditions, with slight differences between breeds. In MON, the genetic correlations between trait levels under current conditions and slopes at high THI were strongly negative for PY and rather positive for CR. Therefore, in this breed, the cows with the best CR in current conditions would be among those with CR that would be the least affected by heat stress in future conditions. The correlations between PY and CR slopes tended to be positive, indicating that the response to heat stress could affect both traits similarly. These slopes can therefore be interpreted as indicators of adaptation, even if their variability is limited.

## Data Availability

The phenotypic and pedigree data originated from the French National Animal Breeding database used for selection purposes. Data are owned by French farmers and restrictions apply regarding their availability. The Safran meteorological database can be accessed through Meteo France for research purposes.
